# Relationship between myocardial scar and hypertrophy by LGE CMR in hypertrophic cardiomyopathy patients with and without clinical events

**DOI:** 10.1186/1532-429X-14-S1-P165

**Published:** 2012-02-01

**Authors:** Aya Kino, Lubna Choudhury, James Carr

**Affiliations:** 1 Radiology, Northwestern University, Chicago, IL, USA; 2 Department of Medicine, Cardiology Division, Northwestern University/Feinberg School of Medicine, Chicago, IL, USA

## Summary

The purpose of this study was to investigate the relationship between the extent of hyperenhancement and left ventricle maximal wall thickness (mWT) detected by LGE CMR and clinical events of nonsustained ventricular tachycardia (NSVT), implantation of cardioverter defibrillator (ICD) or diastolic heart failure in HCM patients that underwent LGE CMR.

## Background

Late gadolinium enhancement cardiac magnetic resonance (LGE CMR) imaging has been used to detect myocardial hypertrophy and scar/fibrosis in Hypertrophic cardiomyopathy (HCM) patients. The presence of hyperenhancement has been associated with progressive ventricular dilation, ventricular arrhythmias and clinical risk factors for sudden cardiac death.

## Methods

Under IRB approved protocol a total of 82 HCM patients underwent LGE CMR images using a 2D PSIR TurboFLASH protocol after administration of 0.2 mmol of gadopentetate dimeglumine per kilogram of body weight. The presence of LGE was assessed using automated software: percentage of scar and maximal wall thickness were calculated. Percentage of scar was compared between patients with mWT <2.5 cm and ≥2.5cm The mean values of percentage scar and mean maximal wall thickness between patients with/ without clinical events (presence of NSVT, placement of ICD or development of diastolic heart failure) were compared.

## Results

Scar was detected in 74.5 % patients and clinical events were present in 54 (%) patients. Only one patient without scar had NSVT and had ICD implanted. There was a significant difference between mWT in patients with scar (2.0 cm) and no scar (1.5 cm) p =0.002. Scar % and mean mWT were significant higher in patients with positive clinical events(Table 1.There was no significant difference between % of scar in patients with risk factors when compared the mWT ≥2.5cm and <2.5 cm. However patients with positive clinical events with mWT≥2.0cm presented higher scar % compared to patients with positive clinical events with mWT<2.0cm (Table2).

**Table 1:** Relationship between % SCAR/ mean wall thickness in HCM and clinical events

	Clinical events +	Clinical events -	p value
%SCAR	30.49	17.26	0.001
Mean Maximal wall thickness (cm)	2.087	1.723	0.006

**Table 2:** Comparison between Scar % and clinical events with mWT of 2.0 or 2.5 cm

Clinical events +			p value
	mWT<2.0cm	mWT≥2.0cm	

% SCAR	25.21	36.16	0.006

	mWT <2.5cm	mWT≥2.5cm	

% SCAR	32.38	34.66	0.71

## Conclusions

In conclusion, HCM patients with a mWT above 2.5 cm maybe at increased risk of events regardless of the amount of myocardial scar.

## Funding

None.

